# Supplementary material for: Negative feedback couples Hippo pathway activation with Kibra degradation independent of Yorkie-mediated transcription
Source: eLife. 2021 Feb 8;10:e62326. doi: 10.7554/eLife.62326 (PMC7895526; doi:10.7554/eLife.62326)
Supplement: Supplementary file 1. — Note the highlighted G residue in KibS677A For primer corresponds to the substitution that will result in the mutation of serine-677 to alanine. [file elife-62326-supp1.docx]

**Supplementary file 1**

| **Primers** | **Sequence** |
| --- | --- |
| Gibson to pUbi_For | TTCTTCCCGCAGATAATCCAAATCGTTAACAGATCTGCGG |
| Gibson to pUbi_Rev | AAGTAAGGTTCCTTCACAAAGATCC |
| Gibson to pMT_For | TCAGTGCAACTAAAGGGAATTCGATATCTCGTTAACAGATCTGCGG |
| Gibson to pMT_Rev | AGGTCGACTCTAGAGGATCCCCGGGAAAGATCCTCTAGAGGTTACTTG |
| ΔWW1 For | AGCAACAACACCACAGCGACTGCTACACAAAGCCGCAGACTTT |
| ΔWW Rev | CTTTGTGTAGCAGTCGCTGTGGTGTTGTTGCT |
| ΔWW2 For | GACTTTCGAGGATTGTGTGGGCGAGTGGAAGACTGTCCAGGAGCA |
| ΔWW2 Rev | TCTTCCACTCGCCCACACAATCCTCGAAAGTCT |
| ΔWW 1&2 For | AGCAACAACACCACAGCGACGAGTGGAAGACTGTCCAGGAGCA |
| ΔWW 1&2 Rev | TCTTCCACTCGTCGCTGTGGTGTTGTTGCT |
| ΔCC1 For | TCGATGAGTCGCCACGATCCGTACACGGAACGGGGCATGAACA |
| ΔCC1 Rev | GTTCCGTGTACGGATCGTGGCGACTCATCGAA |
| ΔCC2 For | ACCTGAACGGAGGAGCCCGTTTCTCGGAGAGCACCTTCTCCATTAGCAGT |
| ΔCC2 Rev | TGCTCTCCGAGAAACGGGCTCCTCCGTTCAGGT |
| 484-1288 For | TTCGTTAACAGATCTGCGGCCGCGCCACCATGAGTAAGAGCGCCTTGAGCTTCAC |
| 484-1288 Rev | TCGCCCTTGCTCACGCCGGAGCCGGTACCGGACACCTCCACGCCGTAGTTGCGA |
| 1-857 For | TTCGTTAACAGATCTGCGGCCGCGCCACCATGCCGAATCTGCAACAAACCGC |
| 1-857 Rev | TCGCCCTTGCTCACGCCGGAGCCGGTACCGGACTCATCCGACGACTCCTCCCGGTTG |
| 858-1288 For | TTCGTTAACAGATCTGCGGCCGCGCCACCATGTCCACCATTACATCCTCCCAGAC |
| 858-1288 Rev | TCGCCCTTGCTCACGCCGGAGCCGGTACCGGACACCTCCACGCCGTAGTTGCGA |
| KibS677A For | CCGGCGATGCTGGCGTCTTCGAG |
| KibS677A Rev | CCACGGATTCATTGCTGACCGC |
| dsRNA hpo 3’-UTR For | TAATACGACTCACTATAGGGAGAGCAACTCACAATTTCGCAAG |
| dsRNA hpo 3’-UTR Rev | TAATACGACTCACTATAGGGAGAATGCTTTCGTGCTGGAAGAT |
| dsRNA wts 3’-UTR For | TAATACGACTCACTATAGGGAGATGGAAATCGAACCTTTCTGG |
| dsRNA wts 3’-UTR Rev | TAATACGACTCACTATAGGGAGATCGTGGGGCTAAACAATTTC |
| dsRNA slimb For | TAATACGACTCACTATAGGGAGAGCACAGGCCTTCACAACCACTATG |
| dsRNA slimb Rev | TAATACGACTCACTATAGGGAGATTGCAGACCAGCTCGGATGATTT |
